# Cell Type-Specific Interferon-γ-mediated Antagonism of KSHV Lytic Replication

**DOI:** 10.1038/s41598-019-38870-7

**Published:** 2019-02-20

**Authors:** Mi-Kyung Park, Hyejeong Cho, Seong Woon Roh, Seong-Jun Kim, Jinjong Myoung

**Affiliations:** 1 Korea Zoonosis Research Institute, Chonbuk National University, Iksan-Si, Chollabuk-do 54531 Republic of Korea; 2 School of Food Science and Food and Bio-industry Research Institute, Kyungpook National University, Daegu, 41566 Republic of Korea; 3 World Institute of Kimchi, Gwangju, 61755 Republic of Korea; 4 Center for Convergent Research of Emerging Virus Infection, Korea Research Institute of Chemical Technology, Yuseong, Daejeon, 34114 Republic of Korea

## Abstract

Kaposi’s sarcoma-associated herpesvirus (KSHV) is causally associated with several malignant tumors: Kaposi’s sarcoma (KS), multicentric Castleman’s disease (MCD), and primary effusion lymphoma (PEL). KS remains the most common AIDS-related malignancy since the AIDS epidemic and thus has been extensively studied. KS is characterized as an angioproliferative disease with massive immune cell infiltration at the early stage. High levels of proinflammatory cytokines and growth factors are found in KS lesions, and their involvement in the survival and growth of tumor cells has been well characterized. However, little is known about the role of the inflammatory microenvironment in the regulation of KSHV gene expression and/or viral replication. In the present study, we demonstrated that IFN-γ and TNF-α profoundly inhibited KSHV progeny production in primary human lymphatic endothelial cells (LECs) as well as induced KSHV-producer cells (iSLK.219) with doxycycline. Of note, IFN-γ inhibited overall KSHV gene expression, while the effects of TNF-α were confined to a selected set of genes, which were also downregulated by IFN-γ. The addition of IFN-γ up to 36 hr after induction of viral lytic replication was effective in terms of the inhibition of infectious virion production, suggesting that its inhibitory effect is exerted at the early stages of KSHV life cycle. We believe these data have potentially important implications for rationalizing a therapeutic agent to treat KSHV-induced tumors in which lytic replication plays a critical role in their pathogenesis: KS and MCD.

## Introduction

Kaposi’s sarcoma-associated herpesvirus (KSHV) belongs to the human herpesvirus γ-subfamily along with Epstein-Barr virus (EBV). The primary infection targets *in vivo* include endothelial and B cells, causing various malignancies, especially in immunodeficient hosts: Kaposi’s sarcoma (KS), primary effusion lymphoma (PEL), and a subset of multicentric Castleman’s disease (MCD). Among KSHV-infected disorders, KS has been the main focus of study in the KSHV field, as it is the most common AIDS-related malignancy, and *in vitro* infection models are available. Although it bears the word sarcoma in its name, KS may be a misnomer^1^, as it is clearly distinct from classical sarcomas in many ways: (1) KS lesions contain many different cell types (^2–4^ and reviewed in^5,6^), unlike traditional sarcomas, which mainly consist of a single cell type; (2) immune infiltrates as well as the tumor elements (so-called “spindle cells”) in the lesions produce a wide variety of proinflammatory and angiogenic products (^7^ and reviewed in^8^); (3) the growth of spindle cells *in vitro* depends generally on the presence of exogenous growth factors such as cytokines and growth factors^9,10^. Therefore, it has been suggested that KS has features of a reactive hyperplasia or inflammatory angioproliferative process, which is a clear distinction from classical sarcomas. In line with this notion, an array of mRNAs encoding cytokines and growth factors are detected in KS lesions, including TNF-α, TNF-β, IFN-γ, IL-1β, IL-6, platelet-derived growth factor (PDGF), basic fibroblast growth factor (bFGF) and granulocyte-macrophage colony-stimulating factor (GM-CSF) (^11–13^ and reviewed in^9^). In addition, KS spindle cells generate a variety of cytokines and growth factors upon *in vitro* explant culture, such as vascular endothelial growth factor (VEGF), bFGF, IL-8, IL-1β and IL-6^14–18^. Of note, *in vitro* growth of KS spindle cells entails the use of conditioned media from activated lymphocytes^19,20^, which contain high levels of IL-6, TNFs, IFN- γ, IL-1β, and oncostatin M (OSM). Thus, one may envision that these proinflammatory cytokines present in the lesions play a central role in the KS pathogenesis. Indeed, IL-1β and PDGF were identified as major mitogens for the spindle cells *in vitro*^12,21^. VEGF, upregulated by several KSHV gene products, is a prominent angiogenic factor, so it is likely responsible for neovascularity and edema in affected tissues^22,23^. bFGF is highly expressed in KS lesions and is known to promote spindle cell growth in an autocrine and paracrine pattern *in vitro*^7^. Furthermore, cytokines produced from activated T cells seem to be responsible for phenotypic and functional changes in endothelial cells relevant to the pathogenesis of KS^24^. Therefore, it is clear that many of these proinflammatory and angiogenic factors are involved in the initiation and growth of KS tumors.

What has remained unclear is whether inflammatory signals can modulate KSHV gene expression and/or replication^25^. In the present study, to shed light on the potential roles of proinflammatory cytokines and growth factors on KSHV lytic replication, we first examined the identities of proinflammatory and growth factors differentially expressed in the conditioned medium (CM) from the human tonsillar explant culture. Among them, IFN-γ and TNF-α had a profound inhibitory effect on infectious virion production in primary human LECs as well as a KSHV-producer cell line (iSLK.219). Interestingly, inhibitory mechanisms of IFN-γ and TNF-α seem to be distinct: IFN-γ inhibits overall viral transcription, while TNF-α does not. Furthermore, IFN-γ seems to act early in the virus life cycle because the addition of IFN-γ up to 36 hr post-induction of viral lytic replication is effective enough to reduce the number of infectious viruses in the culture supernatant. Notably, both IFN-γ and TNF-α inhibit viral replication in LECs but not in human umbilical vein endothelial cells (HUVECs) or hTERT-immortalized microvascular endothelial cells (TIME). Taken together, our data strongly suggest that proinflammatory cytokines (IFN-γ and TNF-α) can modulate KSHV replication in a cell type-specific manner. Further delineation of the underlying molecular mechanisms will provide a rationale to develop effective anti-viral therapeutics against KSHV-induced endothelial pathology.

## Results

### IFN-γ and TNF-α impair KSHV progeny production

**Figure 1:**
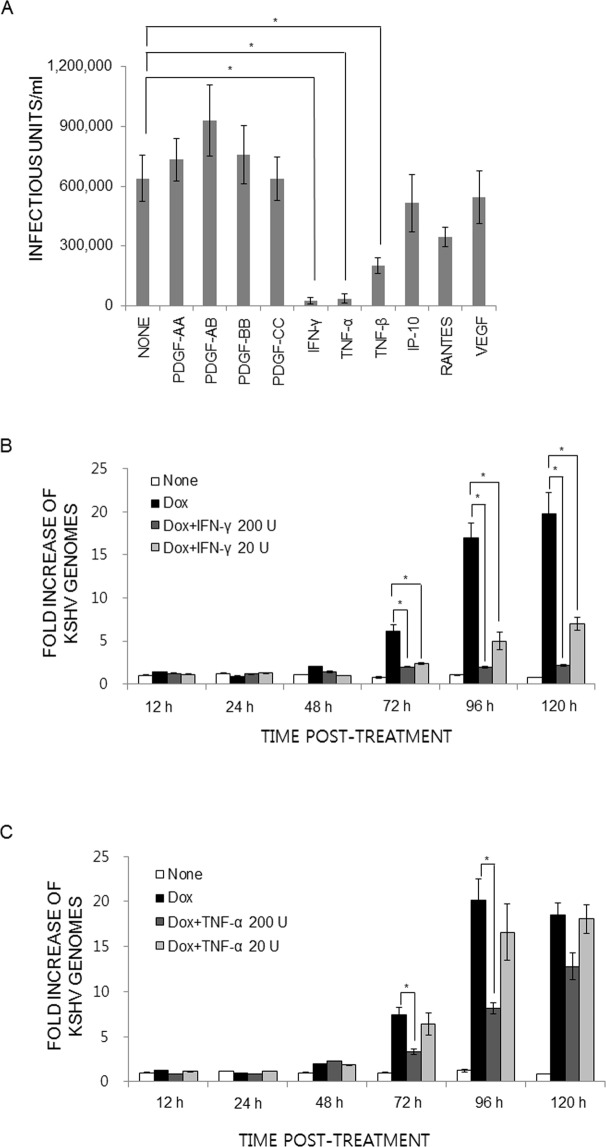
IFN-γ and TNF-α greatly inhibit KSHV progeny production. (**A**) iSLK.219 cells were induced with Dox (0.2 μg/ml), and at the time of induction, each indicated cytokine/chemokine/growth factor was added to the culture. RANTES: 20 units/ml; IP-10, IFN-γ, TNFs, PDGF isotypes: 200 units/ml. At 3 days of treatment, infectious virus titer in the culture supernatant was determined. The means ± SEM of 5 independent experiments performed in duplicate are plotted. For the analysis of KSHV genome replication, iSLK.219 cells were induced as described in (**A**). At the time of induction, IFN-γ (**B**) or TNF-α (**C**) was added into the culture at 20 or 200 units/ml. At the indicated time points, cells were harvested and genomic DNA was prepared for quantitation of the number of the KSHV genomes by real-time PCR. Data are presented as means ± SEM from three independent experiments. *P < 0.01 by one-way ANOVA Tukey Method.

**Figure 2:**
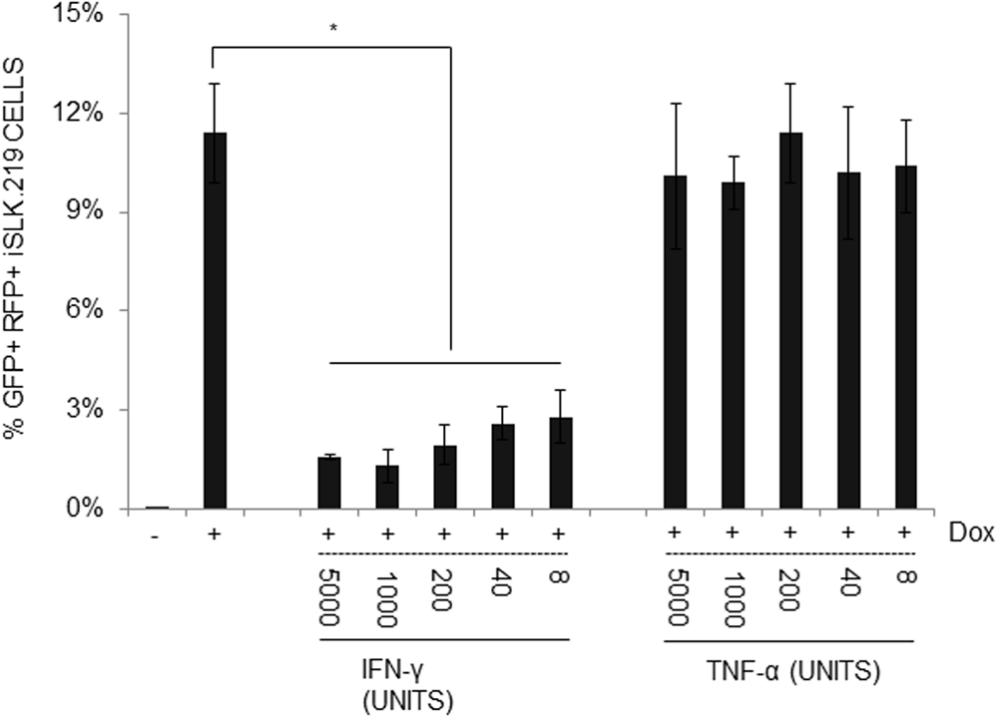
IFN-γ inhibits RFP expression, a lytic marker of KSHV lytic replication, in a dose-dependent manner. iSLK.219 cells were induced without or with Dox (0.2 μg/ml) in the absence/presence of IFN-γ or TNF-α (final 8 to 5000 units/ml) for 2 days before flow cytometry. Live cells were gated for RFP, whose expression was driven by the polyadenylated nuclear (PAN) RNA promoter. Data are presented as mean ± SEM from three independent experiments. *P < 0.01 by one-way ANOVA Tukey.

The roles of proinflammatory cytokines found in KS lesions have mostly been studied in primary effusion lymphoma (PEL)-derived cells such as body cavity-based cells (BCBL-1), BC-1, or BC-3. A few cytokines and chemokines induce the expression of selected sets of lytic genes in treated PEL cell lines. However, those immune-modulatory cytokines/chemokines/growth factors are found in the lesion, and infiltrating mononuclear cells are not infected. Therefore, it is possible that primary targets of proinflammatory cytokines are endothelial cells and endothelial cell-derived spindle cells. Few studies have analyzed the effects of those cytokines/chemokines on the primary target of KSHV infection: endothelial cells. To study the roles of cytokines/chemokines on KSHV lytic replication, we first sought using 64-plex ELISA the identities of cytokines, chemokines, or growth factors in the culture supernatants of tonsillar cells (see Materials and Methods). Briefly, 1 × 10^6^ KSHV-infected or naïve B cells were co-cultured for two days with 1 × 10^6^ naïve B or T cells with or without phytohemagglutinin (PHA) stimulation. Culture supernatants were harvested and analyzed for levels of cytokines/chemokines/growth factors. A selected set of them are summarized in Table 1. Of note, expression levels of one of the two chemokines (IP-10), three cytokines (IFN-γ, TNF-α, TNF-β), and one growth factor (platelet-derived growth factor A homo-dimer, PDGF-AA) were ca. 50% higher in the culture supernatants from the co-culture of infected B cells and PHA-stimulated T cells than those from PHA-stimulated T cell alone, implying KSHV infection in tonsillar B cells may modulate immune-regulatory molecules. To examine effects of these cytokines/chemokines/growth factors on KSHV replication in iSLK.219 cells, a Dox-inducible KSHV-harboring SLK cell line^26,27^, cells were induced with Dox (0.2 μg/ml) and treated with each indicated molecule at the time of induction (Fig. 1). To analyze the different effects of PDGF isoforms, various homo- or hetero-dimers of PDGF were investigated as well. As vascular endothelial growth factor (VEGF) is expressed at high levels in KSHV lesions, it was included in this study as well, although its level in the conditioned medium was low (data not shown). At d 3 post-induction, infectious virus titers in the culture supernatants were determined (Fig. 1A). Notably, IFN-γ and TNF-α inhibited infectious virus production by over 90%, while TNF-β and IP-10 displayed moderate (50~60%) inhibition on KSHV lytic reactivation. Interestingly, KSHV genome replication was severely impaired by IFN-γ at both high and low concentrations (Fig. 1B). In addition, the differences in inhibitory activity between the two doses of IFN-γ were statistically significant (*P* < 0.01 at 96 and 120 hr post-treatment). The inhibitory effect of TNF-α on viral DNA synthesis was initially apparent. However, it largely disappeared at later stages of viral replication (Fig. 1C). As IFN-γ and TNF-α demonstrated a strong inhibitory activity on infectious virus production in iSLK.219 cells, we hypothesized that expression of lytic genes may be negatively affected by the treatment of those cytokines. As rKSHV.219 harbors a RFP expression cassette, expression of which is driven by a KSHV lytic gene promoter, levels of RFP expression in the presence/absence of IFN-γ or TNF-α at varying concentrations were examined (Fig. 2). Without induction with Dox, few cells were positive for RFP. At d2 post-induction, ca. 12% cells expressed RFP, indicating those cells are lytically activated. Of note, when iSLK.219 cells were treated with Dox and IFN-γ (5000 units/ml), RFP expression was decreased by 86% compared to that of the Dox alone control, and even at the lowest IFN-γ concentration (8 units/ml), RFP expression was reduced by 71%. Interestingly, TNF-α treatment did not show any reduction of RFP expression, implying that the mechanisms of those two cytokines are likely to be different.

**Table 1:** Expression of cytokines, chemokines, and growth factors from co-cultures of tonsillar B and T cells.

Co-culture		IP-10	RANTES	IFN-γ	TNF-α	TNF-β	PDGF-AA
Infected	Uninfected						
(unit: ng/ml^§^)							
B	none	ND	0.1	0.4	ND	0.3	0.1
B	B	ND	2.0	2.9	0.2	1.0	0.1
none	T w/o PHA*	3.7	54.1	2.4	8.4	3.3	1.7
none	T w/PHA^#^	3.9	189.5	7.1	22.5	5.3	4.1
B	T w/o PHA	4.8	48.2	2.6	6.5	1.5	0.4
B	T w/PHA	6.7	190.5	12.8	38.8	7.3	6.9

The mean of duplicated samples is shown.

*T cells not stimulated with phytohaemagglutinin (PHA);

^#^T cells stimulated with PHA for 6 hr prior to co-culture with B cells; ND, not detected.

^§^Specific activity of each cytokine was not measured. For comparison, specific activities of recombinant proteins utilized in this study are provided. RANTES: 2 × 10^5^ units/mg; IP-10, IFN-γ, TNFs, PDGF isotypes: 2 × 10^7^ units/mg.

### IFN-γ and TNF-α inhibit infectious KSHV virion production in primary human LECs

**Figure 3:**
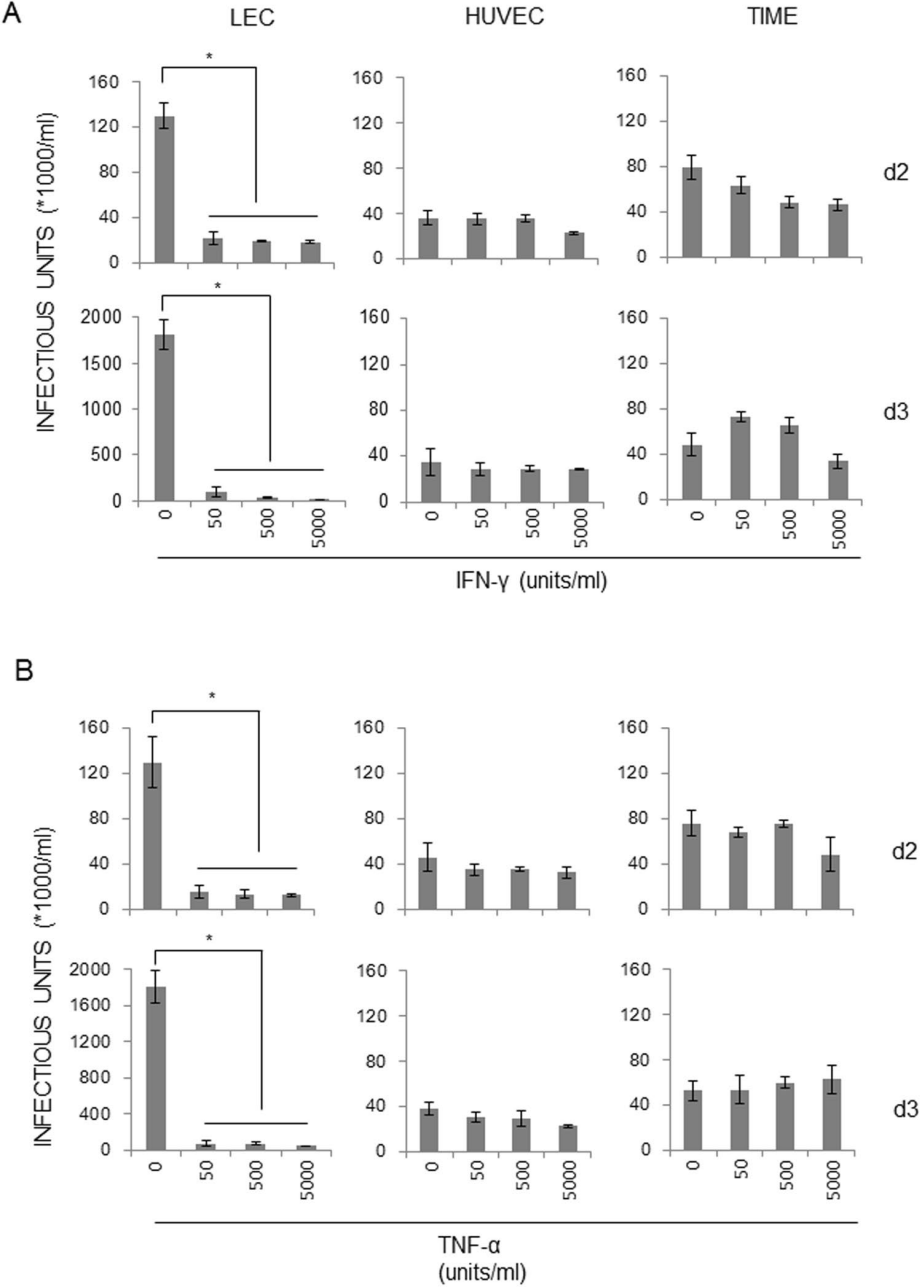
IFN-γ and TNF-α impair the production of infectious viruses in LECs. Human primary cells (LECs, HUVECs, TIME) were infected with rKSHV.219 at MOI = 3 in the absence/presence of IFN-γ (**A**) or TNF-α (**B**) (0 to 5000 units/ml) for the indicated time period. Infectious viruses in the culture supernatant were determined. The means ± SEM of three independent experiments is plotted. *P < 0.01 by one-way ANOVA Tukey Method.

As IFN-γ concentration was determined to be 250 units/ml in the conditioned medium of the co-culture of infected B and stimulated T cells (Table 1), we hypothesized that T cell-derived IFN-γ may have a profound effect on KSHV replication in endothelial cells. As SLK cells turned out to be an epithelial cell line^28^, confirmation of IFN-γ-mediated inhibition of KSHV replication in primary endothelial cells is of paramount importance. LEC’s have been suggested to be the most physiologically relevant target *in vivo*^29^. Therefore, three different primary human cells were employed for comparisons: LECs, HUVECs, and human telomerase reverse transcriptase (hTERT)-immortalized microvascular endothelial cells (TIME). Primary endothelial cells were infected with rKSHV.219 at MOI = 10, and unbound viruses were extensively washed off before the addition of IFN-γ (Fig. 3A) and TNF-α (Fig. 3B) in the culture at varying concentrations. Infectious virus titers in the culture supernatant were determined at d 2 (top panels) or d 3 post-infection (bottom panels). Notably, only LECs, a likely infection target *in vivo*^29,30^, demonstrated IFN-γ-mediated inhibition, while HUVEC and TIME did not, suggesting the biological and physiological importance of IFN-γ *in vivo*.

### The different responses of primary endothelial cells to IFN-γ and TNF-α are not related to the expression levels of cognate cytokine receptors on the cell surface

**Figure 4:**
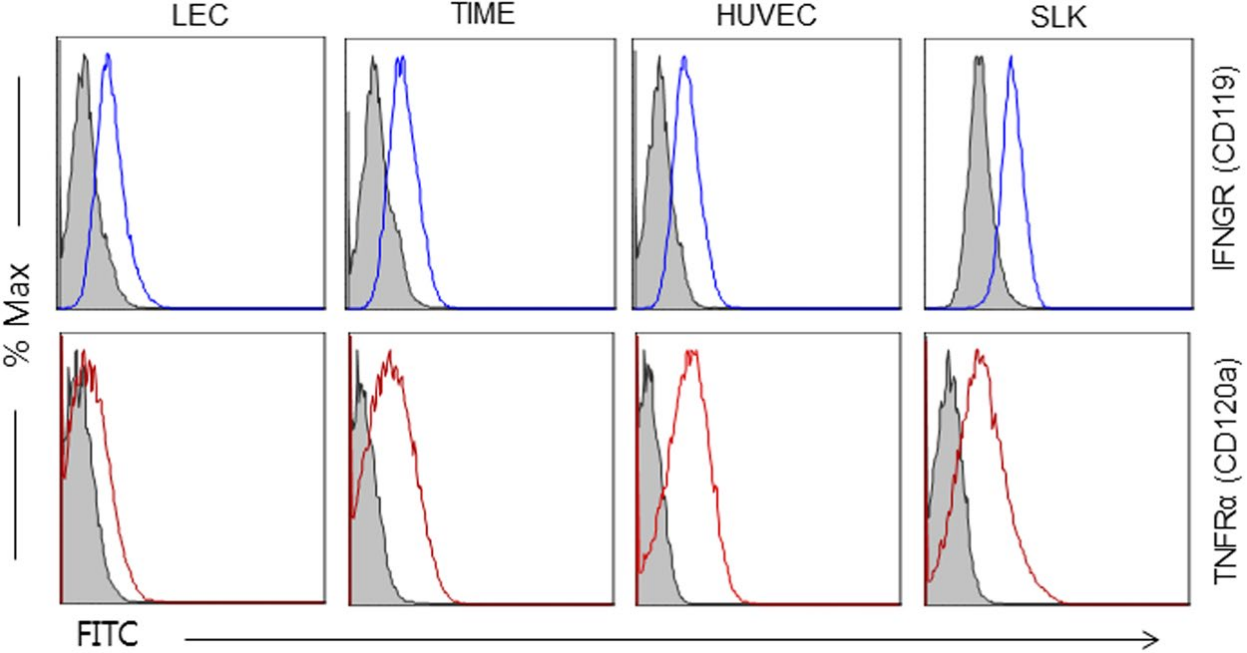
Levels of the surface expression of IFN-γ and TNF-α receptors are comparable between primary and transformed human cells used. The expression of IFNGR (CD119) and TNFRα (CD120a) was assessed by flow cytometry in the indicated cell type using specific antibodies. Filled and open histograms represent isotype and each receptor-specific antibody staining, respectively. One representative result is shown from 3 independent experiments.

To investigate the basis of the different outcomes of cytokine treatment on different cell types, the expression of the ligand-binding polypeptide chain of their cognate receptors on the cell surface was assessed by flow cytometry using specific antibodies (Fig. 4). Interestingly, levels of the cytokine receptors (IFN-γ receptor 1 (IFNGR1 (CD119)) and TNF-α receptor α chain (TNFRα (CD120a)) were comparable among all the cell types examined: LECs, HUVECs, TIME, and SLK cells. These data suggest that the differences in the capacity and/or scope of intracellular signaling upon ligand binding on the surface are likely responsible for the different outcomes of cytokine treatment.

### IFN-γ inhibits early stages of KSHV lytic replication

**Figure 5:**
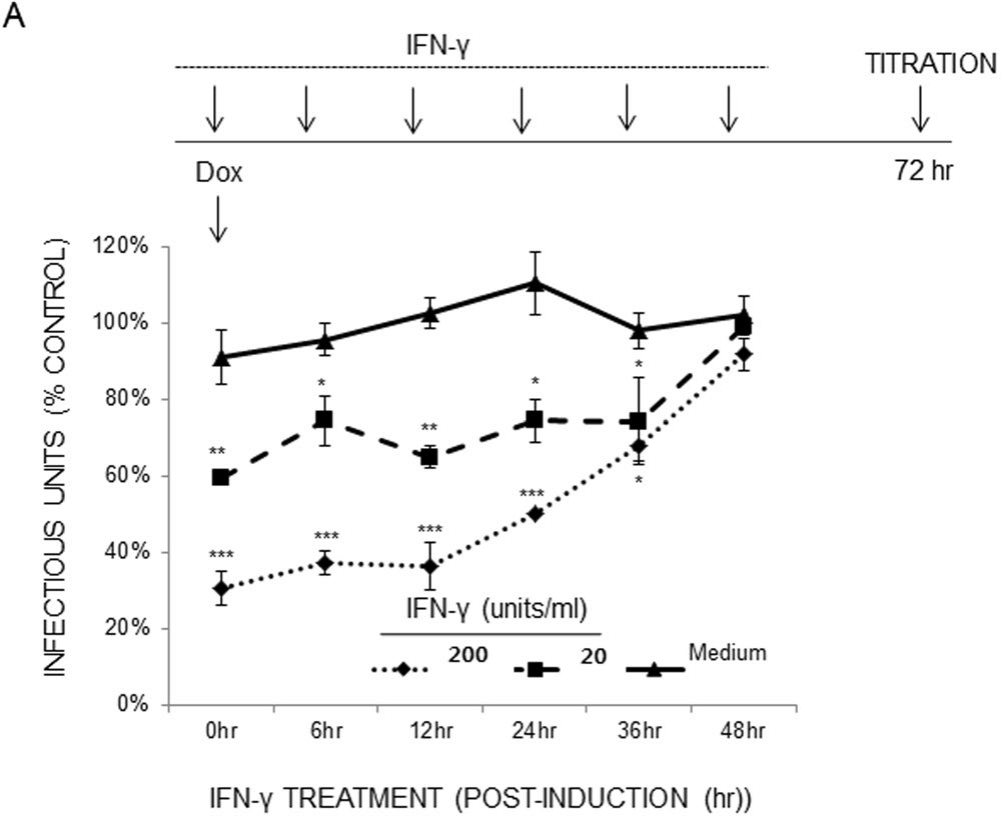
IFN-γ inhibits KSHV lytic replication in a time- and dose-dependent manner. iSLK.219 cells were induced without or with Dox (0.2 μg/ml), and IFN-γ (0, 20, or 200 units/ml) was added into the culture at the indicated time. Infectious virus titer in the culture supernatant was determined at 72 hr post-induction and normalized to the medium-treated control. The means ± SEM of triplicated samples is plotted. One representative result is shown from 3 independent experiments. *P < 0.01 by one-way ANOVA Tukey method.

To investigate which stages of viral replication are affected by IFN-γ, iSLK.219 cells were induced with Dox (0.2 μg/ml), and IFN-γ (0, 20, 200 units/ml) was added into the culture at varying time points. At 72 hr post-induction, culture supernatants were collected for the evaluation of infectious titer (Fig. 5). When IFN-γ was administered up to 36 hr post-induction, KSHV progeny production was significantly inhibited in a dose-dependent manner. As it takes usually 48 hr before any viruses are detectable in the culture supernatant of iSLK.219 cells upon induction^27^, it is reasonable to conclude that IFN-γ inhibits early stages (immediate-early or delayed-early) of KSHV lytic replication.

### The effect of IFN-γ treatment on viral transcriptome and protein expression

**Figure 6:**
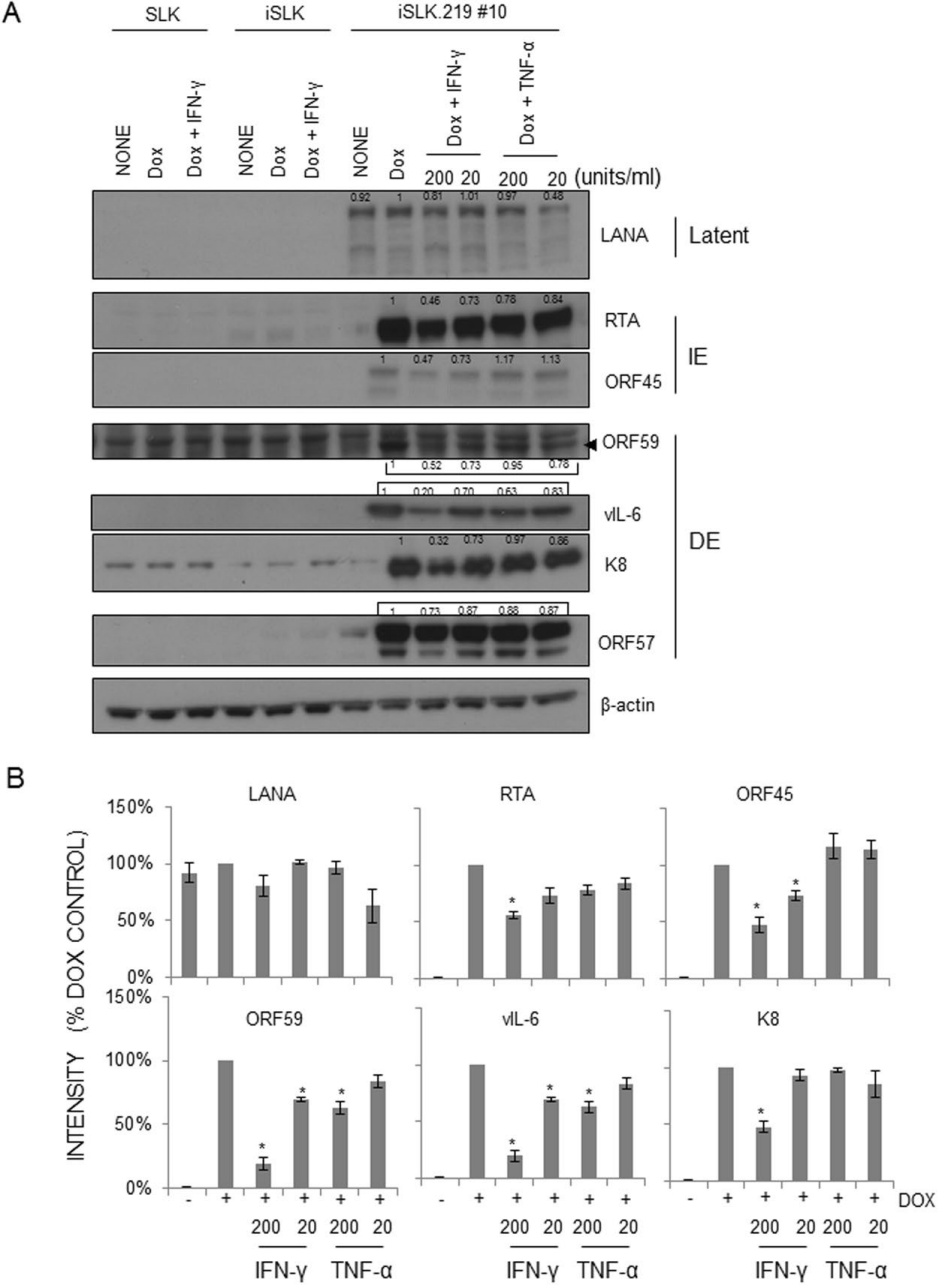
IFN-γ and TNF-α have different effects on KSHV gene expression in a dose-dependent manner. (**A**) SLK, iSLK, and iSLK.219 cells were left untreated or induced with Dox (0.2 μg/ml), and at the time of induction, where indicated, IFN-γ or TNF-α (0, 20 or 200 units/ml) was added to the culture. At 2 days post-induction, cell lysates were prepared for Western blot analysis. Temporal expression of KSHV genes was examined: latent, immediate-early (IE), and delayed-early (DE). Arrowhead indicates the specific ORF59 band. β-Actin was included as a loading control. One representative result is shown from 3 independent experiments. The intensity of each specific band was determined using ImageJ software and was normalized to that of the Dox-treated sample. The numbers indicate the relative intensity compared to the Dox-alone control. (**B**) The means ± SEM of 3 independent experiments are plotted. *P < 0.01 by one-way ANOVA Tukey Method.

**Figure 7:**
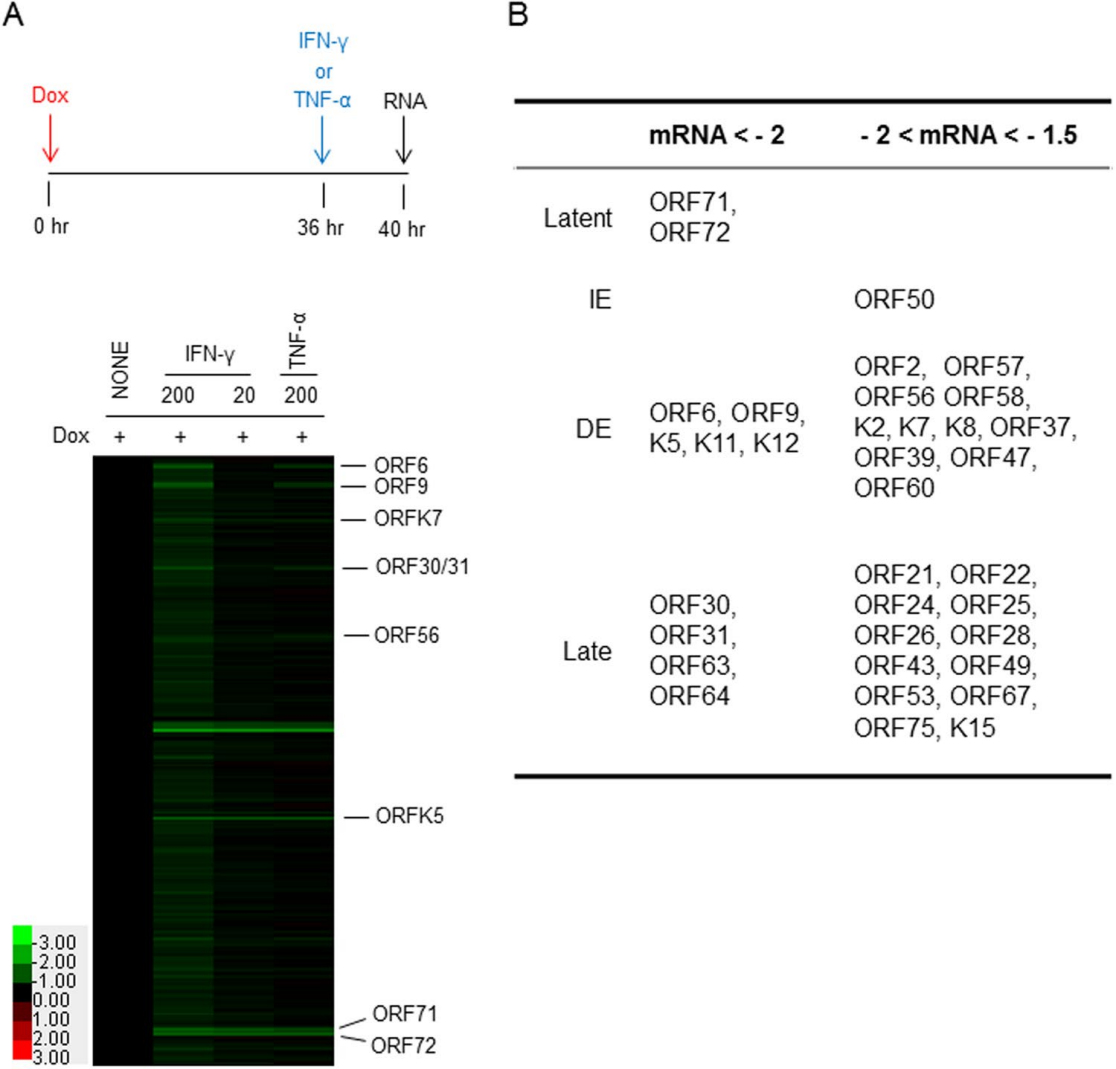
IFN-γ globally down-regulates viral gene expression, but TNF-α does not. (**A**) iSLK.219 cells were induced to lytic replication by Dox (0.2 μg/ml) for 36 hr before the addition of IFN-γ or TNF-α. The concentration of each cytokine is indicated (units/ml). Four hours after treatment with the cytokines, total RNA was extracted and processed for the custom KSHV tiling array. KSHV genes downregulated by more than 50% by both IFN-γ and TNF-α are indicated on the right. Green bands with strong signal, which are not annotated in the middle of the array, are transcripts from the repeated regions of the one end of the KSHV genome. The scale color bar is shown on the left. (**B**) Summary of KSHV genes that were significantly downregulated by IFN-γ compared to the Dox-alone control. Viral genes that were inhibited by more than 2-fold are shown on the left, and those that were between 1.5- and 2-fold are on the right. Viral genes are grouped according to their temporal regulation. IE: immediate-early genes; DE: delayed-early genes.

To analyze how IFN-γ and TNF-α affect viral gene expression, SLK, iSLK, and iSLK.219 cells were left uninduced or induced with Dox (0.2 µg/ml), and at the same time, IFN-γ or TNF-α was added in the culture at the indicated concentration (0, 20, or 200 units/ml). At d 2 post-induction, cell lysates were prepared for Western blot analysis. Various viral proteins were blotted using specific antibodies: LANA (latent), RTA and ORF45 (immediate-early), ORF59, vIL-6 (K2), KbZIP (K8), and ORF57 (delayed-early). Of note, IFN-γ inhibited viral gene expression in a dose-responsive manner, especially RTA, ORF59, and K8, all of which play important roles in viral DNA synthesis (Fig. 6). Apparently, TNF-α had little, if any, effect on viral gene expression, which is consistent with normal RFP expression driven by RTA (Fig. 2). To investigate if IFN-γ-mediated inhibition of viral protein expression is at the transcriptional level, Dox-induced iSLK.219 cells were treated with each cytokine at 36 hr post-induction. The time point was selected based on the data in Fig. 5, because infectious virus particle production was sensitive to cytokine treatment up to that time point. At 4 hr post-treatment, total RNA was extracted for microarray analysis of the viral transcriptome (Fig. 7A). Notably, IFN-γ inhibited overall viral gene transcription in a dose-dependent manner while TNF-α-mediated inhibition was evident only in a limited number of viral genes. Moreover, several genes were regulated by both IFN-γ and TNF-α, most of them immediate-early or delayed-early genes (RTA; lytic switch protein, ORF6; DNA-binding protein, ORF9; DNA polymerase, ORF56; DNA replication protein, K5 & K7; regulators of cellular proteins). This is in line with the notion that IFN-γ regulates early stages of viral replication (Fig. 5). Interestingly, among genes that were affected by both IFN-γ and TNF-α were ORF K13 (vFLIP) and ORF 72 (vCyclin D). Currently, studies are underway to analyze the biological significance of cytokine-mediated reduction of viral gene expression in the course of KSHV lytic replication. The identities and levels of significantly affected genes are summarized in Fig. 7B^31,32^.

## Discussion

KS is the most common AIDS-related malignancy since the AIDS epidemic in the early 1980’s. It is characterized by extensive angiogenesis, spindle cell proliferation, and massive immune cell infiltration. High levels of proinflammatory cytokines are detected in KS lesions, and their roles in the KSHV-induced pathologies have been postulated to help in the proliferation of tumor elements and recruitment of naïve cells for viral spreading and thus the initiation and/or development of KS lesions (reviewed in^6,8^). However, their involvement in the regulation of viral replication remains undefined. It is worth noting that Parravicini *et al*.^33^ analyzed expression of 4 KSHV proteins (LANA, vIRF1, vIL-6 and PF-8) by immuno-histochemistry, showing that KSHV protein expression is somewhat restricted *in vivo*, while viral lytic proteins are readily detected in KSHV-infected cell lines *in vitro*. On the other hand, some other studies detected expression of more numbers of lytic gene transcripts in at least a subset of AIDS-KS patients with or without anti-retroviral therapies^34–36^, hinting at a tug of war for viral gene expression between viruses and the host. These data suggest that differential protein expression *in vivo* vs *in vitro* may be related to immune restriction of viral replication. To shed light on the roles of proinflammatory cytokines on KSHV replication, we employed human lymphoid aggregate culture (HLAC) prepared from tonsils based on several reasons: (1) KSHV titer is highest in the saliva among all the human body fluids^37^, and some epidemiological studies implicate horizontal transmission as the primary mode of infection by viruses shed in the saliva^38–41^; (2) tonsils are localized in the oral cavity and a rich source of lymphocytes, where activated T cells seem to suppress spontaneous lytic replication in infected B cells, as suggested by *in vitro* studies employing HLAC^42,43^; (3) infiltrating T cells in KS lesions produce an array of cytokines, which support the survival and growth of spindle cells, the tumor element of the lesions^1,9,10^. Thus, we hypothesized that conditioned media from the HLAC culture would also affect the course of KSHV infection. Based on 64-plex ELISA on the conditioned medium (Table 1), a dozen differentially expressed proinflammatory cytokines/chemokines/growth factors were selected and investigated for their ability to regulate KSHV replication. The list of proinflammatory cytokines (Table 1) is largely in line with previous studies (^24,44–48^ and reviewed in^9^). Interestingly, strong inhibition of viral progeny production was observed in induced iSLK.219 and infected LECs treated with IFN-γ- or TNF-α (Figs 1, 3 and 4). Both IFN-γ- and TNF-α are found at high levels in KS lesions (^24,44,49^ and reviewed in^1^). To our knowledge, this is the first report to demonstrate the inhibitory effects of these cytokines on infectious virion production in LECs, the most physiologically relevant cell type for KS of endothelial origin.

One may envision that IFN-γ-mediated antagonism of KSHV lytic replication may seem contrary to the results of previous studies using KSHV-harboring B cell lymphoma cell lines. In fact, several groups have shown that IFN-γ promotes viral lytic gene expression in BCBL-1 or BC-1 cells^50,51^. These discrepancies may be simply explained by cell-type-specific differences in response to proinflammatory cytokines. However, those studies analyzed only selected sets of viral lytic genes, such as RTA, ORF59, or K8.1 at the transcriptional^49–51^ or translational^49,50^ level, and the consequences of the increases in viral mRNA levels were not evaluated in terms of viral progeny production. Of note, Pozharskaya *et al*.^52^ showed that the treatment of IFN-γ (200 units/ml) induced expression of antiviral proteins in BCBL-1 cells, such as double-stranded RNA-activated protein kinase (PKR), 2′5′-oligoadenylate synthetase (2′5′-OAS) and interferon regulatory factor 1 (IRF1), expression of which subsequently led to 60% reduction in the production of infectious virus induced by TPA although IFN-γ indeed stimulated a minor increase in the expression of selected viral lytic genes in the IFN-γ-treated BCBL-1 cells. More detailed and careful studies are warranted to define the roles of proinflammatory cytokines on KSHV replication in KSHV-harboring B cell lymphoma cells. In addition, it remains to be examined if induction of lytic replication by IFN-γ actually takes place in patients with B cell lymphomas, leading to production of infectious viruses *in vivo*. Furthermore, as only a small portion of PEL cell lines in culture are lytically activated *in vitro*, the biological significance of limited lytic replication in B cell lymphomas, even if happening *in vivo*, is questionable. Moreover, PEL cases are mostly monoclonal tumors and thus may not depend on the expression of lytic genes for tumor growth^1,8,9^. However, we cannot formally exclude the possibilities that spontaneous lytic replication occurs *in vivo* in patients with PEL tumors and that lytic gene products promote angiogenesis and tumor growth as some lytic gene products are known to function to that end^22,53^.

The lack of an animal model to analyze KSHV pathogenicity makes it difficult to elucidate the roles of proinflammatory cytokines in the context of viral infection *in vivo*. The role of IFN-γ in the control of viral replication *in vivo* has been studied using a mouse herpesvirus. In murine gammaherpesvirus 68 (MHV68) infections, a close cousin of KSHV, IFN-γ plays a critical role in the regulation of a chronic gammaherpesvirus infection^54,55^ and in the inhibition of reactivation from latency depending on cell types analyzed^56,57^: macrophages were sensitive to IFN-γ-medicated inhibition, but B cells were not. Taken together, these data suggest that IFN-γ seems to regulate gammaherpesvirus replication in a cell type-specific manner.

It is interesting to note that the mechanisms of action of IFN-γ and TNF-α may diverge in terms of RFP expression (Fig. 2), which is driven by the PAN promoter harboring the RTA-responsive element, and in viral gene expression at the transcriptional (Figs 6 and 7) and translational (Fig. 6) levels. IFN-γ treatment led to a sharp decrease in RFP expression, while TNF-α treatment did not (Fig. 2), in spite of its comparable inhibitory activities on infectious KSHV progeny production in transformed (Fig. 1) and primary endothelial cells (Fig. 3). Because extracellular levels of ligand-binding subunits of cytokine receptors were largely comparable on those cells (Fig. 4), it would be reasonable to postulate that differences in intracellular signaling pathways activated by IFN-γ or TNF-α ligation on the cognate receptor of various cell types may lead to differential outcomes of viral gene expression, viral DNA synthesis, capsid assembly, viral exit, or combinations thereof. Hints of their differential mechanisms came from viral protein expression (Fig. 6) and transcriptome analysis (Fig. 7). IFN-γ inhibited important KSHV lytic genes at both the transcriptional and translational levels, such as RTA, ORF45, ORF59, vIL-6 and K8, while TNF-α had little effect on them. Its underlying molecular mechanisms are now being investigated. Notably, the addition of IFN-γ up to 36 hr after induction of lytic replication effectively reduced infectious virion production (Fig. 5) in the culture supernatant, suggesting that IFN-γ inhibits early stages of KSHV replication. In line with this notion, the list of genes downregulated by IFN-γ includes the immediate-early RTA (lytic switch) and the delayed-early ORF6 (single-stranded DNA-binding protein, homologue of EBV Balf2 and HSV-1 ICP8), ORF9 (DNA polymerase), ORF56 (primase), and K8 (KbZIP), all of which are associated with Ori-Lyt-dependent DNA replication (Figs 6 and 7). In a previous report^58^, IFN-γ-mediated inhibition of RTA gene expression is substantiated by reduced RTA promoter activity in transformed dermal microvascular endothelial cells (tDMVEC), suggesting transcriptional regulation of RTA by the cytokine. Furthermore, fold increase of the KSHV genome copy number (Fig. 1B), an indication of initiation of the late stage of KSHV replication, became statistically significant by 48 hr post-induction with Dox: untreated (1 ± 0.02) vs Dox (2.01 ± 0.01) at 48 hr; *p* < 0.01. Taken together, it is reasonable to conclude that IFN-γ inhibits KSHV lytic replication and viral progeny production by interfering with early stages of the virus life cycle, including but not limited to, viral DNA synthesis.

Interestingly, the inhibitory mechanisms of TNF-α seem to be very different from those of IFN-γ, as viral gene expression was in general unaffected by TNF-α treatment at the transcriptional and translational levels, except for a subset of genes (Figs 6 and 7) that were downregulated at the transcriptional level: ORF6, ORF9, ORF30, ORF31, ORF56, ORF K5, ORF71 (vFLIP), and ORF72 (vCyclin). We could not analyze their protein levels due to the lack of good specific antibodies. A few plausible explanations for the relative lack of effect of TNF-α include; (1) TNF-α may modulate viral assembly or exit without much changing the viral transcriptome (Fig. 7) or viral DNA synthesis (Fig. 1C). Some previous studies support this notion as TNF-α did not affect viral gene expression in BCBL-1 cells^51^. (2) TNF-α may downregulate the translation of select viral genes without affecting transcription in general, and some of those affected genes may be indispensable for KSHV lytic replication. Indeed, ORF6, ORF9, and ORF56, which were downregulated by TNF-α, participate in the formation of the replication complex on the lytic origins. (3) TNF-α may activate intracellular host signaling molecules that inhibit various stages of the viral life cycle. For example, TNF-α activates NF-κB signaling, which counteracts KSHV lytic replication and thus promotes viral latency^59–61^. Comparable levels of cognate cytokine receptors on the surface of transformed and primary endothelial cells as well lend support to the possible roles of intracellular signaling molecules activated by TNF-α (Fig. 4). In reality, to some extent, all of the above mechanisms may contribute to the inhibition of viral progeny production by TNF-α. Mechanistic studies are currently underway to define the underlying mechanisms of inhibitory activities of TNF-α on KSHV replication.

Taken together, our data demonstrate that IFN-γ and TNF-α, found at high levels in KS lesions, exert profound inhibitory effects on infectious KSHV virion production. These data suggest that proinflammatory cytokines not only modulate spindle cell proliferation and angiogenesis but also regulate viral replication. Considering the highly lytic nature of *in vitro* KSHV infection in LECs (Fig. 3 and unpublished observations)^29^, it is tempting to postulate by extrapolation that viral infection in endothelial cells *in vivo* may be rendered latent by the inflammatory microenvironment in KS lesions. This is in line with previous histological, virological, and immunological findings that most infected cells in KS lesions are tightly latent^33,62^. Therefore, it would be interesting to determine if these cytokines play a role in pushing spindle cells into latency *in vivo*, as seen in primary endothelial cells *in vitro*. Last but not least, our data provide a rationale to potentiate IFN-γ and TNF-α signaling pathways in infected cells of patients with KSHV-induced tumors, where lytic replication plays an important role in the pathogenesis: KS and MCD. Caution is warranted, though, as pleiotropic cytokines such as IFN-γ and TNF-α affect many different cell types and direct systemic or local administration of them may have unwanted aftermaths^63–65^.

## Materials and Methods

### Cells and reagents

QBI293A cells (Q-Biogene) were used to titrate rKSHV.219 stocks prepared from stable iSLK.219 cells harboring the recombinant virus, which was described before^27,66^. Briefly, parental SLK cells were implanted with a Dox-inducible RTA-expressing system (pRetroX-Tet-On Advanced, Clontech) to generate iSLK cells. rKSHV.219 viruses were infected into iSLK cells, and the resulting cells were selected by puromycin to generate iSLK.219 cells. Primary human LECs, blood endothelial cells (BECs), and HUVECs were purchased from Lonza. Human TERT-immortalized dermal microvascular endothelial cells (TIME) were described elsewhere^67^. LECs, BECs, and TIME cells were cultured in EBM-2 media supplemented with the EGM-2MV BulletKit while HUVEC media were supplemented with the EGM BulletKit. Human lymphoid aggregate culture (HLAC) was prepared and used for this study as described previously^42,68^. SLK cell lines were maintained in DMEM containing 10% fetal bovine serum with supplements as described before^26^ (1% penicillin-streptomycin (Life Technologies) and antibiotics (250 μg/ml G418, 400 μg/ml hygromycin, 10 μg/ml puromycin, 1 μg/ml blasticidin (Invivogen))). Antibiotic-free medium was used when cells were induced to lytic replication with doxycycline (Dox, 0.2 μg/ml). All cytokines, chemokines, and growth factors were purchased from Peprotech except platelet-derived growth factor CC isoform (PDGF-CC, R&D Systems) and resuscitated according to the instructions. Anti-human CD119 (IFN-γRα chain, GIR-94) and anti-human CD120a (TNF-αRα chain) antibodies were purchased from Biolegend.

Antibodies specific to viral proteins were purchased from various vendors; K8 from Santa Cruz, ORF45 and ORF57 from Abcam, β-actin from Sigma-Aldrich, ORF59 from Advanced Biotechnologies. LANA and RTA antisera were custom-made in rabbits.

### Detection of cytokines, chemokines, and growth factors in the culture supernatants from tonsillar cell co-cultures

One hundred microliters of duplicated culture supernatants were used for 64-plex ELISA using the human cytokine array service (Cat # HD64) from Eve Technologies (Alberta, Canada). Raw assay results provided by the company were sorted for molecules over the detection limit and differential expression levels were summarized in Table 1.

### Virus preparation, infection and titration

Virus stocks were prepared from induced iSLK.219 cells as described elsewhere^27,42^. Briefly, induced culture medium was cleared and pelleted by a series of centrifugations, and virus pellets were resuspended in an appropriate cell culture medium. Infectious virus titers of virus stocks and samples were determined in QBI293A cells as previously described^43^. In brief, QBI293A cells were infected with rKSHV.219-containing samples by spinoculation and were cultured for 2 days before subjected to flow cytometry. Virus titers were determined by using the Poisson distribution formula: virus titer = −N × ln (1 − P), where N and P denotes the total number of cells and the proportion of GFP-positive cells in a given well, respectively. Virus stocks were diluted and infected into primary human endothelial cells by spinoculation^43^ at the indicated multiplicity of infection (MOI). Dox-induced iSLK.219 cells or infected human primary endothelial cells were treated with various cytokines, chemokines, or growth factors for the indicated time periods before culture supernatants were collected for virus titration.

### Quantitation of the relative genomic copy number of rKSHV.219 in iSLK.219 cells

Genomic DNA was extracted from cells using DNeasy Blood & Tissue Kit (Qiagen, USA). The relative copy number of KSHV genomic DNA in total DNA was assessed by CFX Connect Real-Time PCR Detection System (Biorad, USA) using the AccuPower 2X GreenStar qPCR Master Mix with primers specific for glyceraldehyde 3-phosphate dehydrogenase (GAPDH) as an internal control and KSHV-encoded latency-associated nuclear antigen (LANA). GAPDH forward: 5′-TCATCCAAGCGTGTAAGGGT-3′; GAPDH reverse: 5′-GGACTGAGATTGGCCCGAT-3′; LANA forward: 5′-AAACAGAAACGGCCAATAACC-3′; LANA reverse: 5′-CCGTAAGGCACCCTTATCTTT-3′. Specificity of amplification was assessed by analyzing melting curve. KSHV genome copy number was normalized by that of GAPDH and the fold increase of the KSHV genomic DNA copy number was calculated compared to that of uninduced iSLK.219 cells.

### Western blot analysis

Western blotting was conducted using NuPAGE SDS-PAGE Gel System (Life Technologies). Cell lysates were prepared in NETN 150 buffer (sodium chloride, 150 mM; EDTA, 2 mM; Tri-HCl, 50 mM; NP-40, 1%) containing protease inhibitor cocktail (cOmplete mini, Roche). Protein separation and Western blotting were performed according to the manufacturer’s instructions^69,70^.

### Flow cytometry

Cells were subjected to flow cytometry analysis with an LSRII (BD Biosciences), and the data were analyzed by FlowJo software. Transformed or primary endothelial cells were trypsinized before staining with specific antibodies to CD119 or CD120a with subsequent washes before being subjected to flow cytometry.

### Custom KSHV tiling array

Total RNA was extracted from induced iSLK.219 cultured in the presence/absence of inhibitory cytokines using RNA-Bee (Tel-Test Inc.) and further purified utilizing the RNEASY Mini Kit (QIAGEN) according to the manufacturer’s instructions. Detailed procedures were described elsewhere^26,42^. Briefly, Cy5 (samples) or Cy3 (reference)-labeled complementary RNA (cRNA) was prepared using the Quick Amp Labeling Kit (Agilent) according to the manufacturer’s protocols. Labeled cRNAs were hybridized onto a custom KSHV tiling microarray (Agilent, custom design identification number: 029238). Array scanning was performed using the GenePix (Molecular Devices), and features were extracted using GenePix Pro software. Heat maps were generated from annotated data using the Java Treeview software.

### Statistics

Data represented in this study are shown as means ± SEM of 3 or more independent experiments. The significance of differences in the mean values was evaluated by one-way ANOVA Tukey method. *P* < 0.01 was considered statistically significant.

## Data Availability

All data generated or analyzed during this study are included in this published article.
